# The ethics conundrum in Recall by Genotype (RbG) research: Perspectives from birth cohort participants

**DOI:** 10.1371/journal.pone.0202502

**Published:** 2018-08-16

**Authors:** Joel T. Minion, Frances Butcher, Nicholas Timpson, Madeleine J. Murtagh

**Affiliations:** 1 Policy, Ethics and Life Sciences (PEALS) Research Centre, School of Geography, Politics and Sociology, Newcastle University, Newcastle upon Tyne, United Kingdom; 2 Oxford School of Public Health, Oxford, United Kingdom; 3 MRC Integrative Epidemiology Unit, School of Social and Community Medicine, University of Bristol, Bristol, United Kingdom; Fred Hutchinson Cancer Research Center, UNITED STATES

## Abstract

**Purpose:**

Recall by genotype (RbG) research recruits on the basis of genetic variation. Increased use of this approach presents an ethical conundrum for cohort studies/biobanks: whether to inform individuals of their genetic information and deviate from standard practice of non-disclosure of results, or mask this information at the level of the individual participant. This paper examines the perspectives of research study participants on RbG research.

**Methods:**

Fifty-three semi-structured interviews were conducted with young adult participants of the Avon Longitudinal Study of Parents and Children (ALSPAC). Topics included understandings of RbG research, expectations around recruitment and communication of research findings.

**Results:**

Participants uniformly expressed a deep trust and faith in ALSPAC and considered themselves part of the ALSPAC team. Such perspectives, alongside a limited knowledge of genetics and modest interest in reported research outcomes, meant few participants reported immediate concerns about being recruited by genotype.

**Conclusion:**

Our findings highlight the responsibility and duty of care on RbG researchers, and longitudinal studies more generally, and the importance of solidarity, reciprocity and co-production in study-participant relations. As such, we consider existing recommendations for conducting RbG research in longitudinal studies in light of our results and speak to recent changes in the approach used by ALSPAC.

## Introduction

Recall by genotype (RbG) studies recruit prospective participants on the basis of genetic variation of interest. This contrasts with more established genetic research methodologies such as genome wide association studies (GWAS), where participants are selected to be representative of specific populations or on the basis of particular phenotypes. The likelihood of participants carrying the genetic variation of interest depends upon the frequency of that variant(s) and the predictability of phenotype manifestation (penetrance). The GWAS approach can be an inefficient and potentially costly recruitment mechanism for rare diseases variants or low frequency variants relevant to common diseases. RbG studies target individuals (or their samples) who have already undergone genotyping. This type of design is able to achieve equivalent biological exposure (i.e. to capture balanced numbers of specific gene variants) and analytical power in a relatively small number of participants as a result of the availability of large pre-genotyped collections. This approach can be effective in dissecting genetic association signals through deep phenotyping in smaller sample sets; it is a cost effective strategy and in the rare variant setting it may be the only feasible approach to recruitment for novel studies or for replication studies [1,2].

RbG studies, however, present novel ethical context and challenges. If participants are to be fully informed of the structure of an RbG study, including naming the variant(s) of interest, then inadvertent disclosure of genetic information during the recruitment process is possible and with it the potential to heighten anxiety among participants about their health status, especially if a variant is implicated in serious or stigmatising conditions. The ethical principle of autonomy (the basis of informed consent in contemporary research) is pitched against the ‘right not to know’ and the principle of non-maleficence (the precept to do no harm) [3]. This central tension in RbG research (also termed Genotype Driven Recruitment/GDR) has been identified [4–6] in studies involving biobanks and other repositories that include participants with genotypic data collection, including disease and tissue-based biobanks [7–9], population-based biobanks^7^ and collections based on health records or direct-to-consumer testing [10]. While such studies have been careful not to conflate the issues of return of clinically useful findings (incidental findings) with disclosure of genetic information through recruitment, they point to a range of cognate issues that bear consideration: the need to avoid participant anxiety if the genetic information–or re-contact itself–is unexpected [4,11]; the possibility that even uncertain information may be important to participants [9]; the potential for over-targeting of rare conditions and thereby potential stigmatisation of such groups; and, the challenge that informational utility includes personal utility and personal meaning as well as clinical utility, especially where there is parental, familial or personal experience of disease [7–9,11]. As a counter to these potential harms, are the harms of *not* pursing the advancement of science through such methods, identified as a human right under Article 27 of the Universal Declaration of Human Rights, i.e. the right “to share in scientific advancement and its benefits” [12].

Empirical studies with participants of disease-based biobanks have also demonstrated participant solidarity [13] with research and researchers, and perceived co-production [14] of research outcomes. Participants, having had long-term relationships with their health care providers and researchers (who may have been one in the same), often felt part of the ‘team’ working towards discoveries and treatments for themselves, their families and the community affected by a disease [7,9]. Conversely, population-based biobank participants have been shown more likely to be anxious about the disclosure of unexpected genetic information [7]. Seven broad recommendations for RbG have been proposed through research participant interviews (mostly disease-based biobank participants), a survey of IRB Chairs and a consensus workshop [15]. While useful and important, these recommendations are context specific and do not work for all circumstances [10]. As noted by Olson and colleagues, guidelines are needed for “all the varied circumstances under which genetic data may become available for researchers” [6]. To this end, we examined what we describe as the ethical conundrum facing RbG studies in the context of a longitudinal birth cohort, the Avon Longitudinal Study of Parents and Children (ALSPAC).

ALSPAC is a “transgenerational prospective observational study investigating influences on health and development across the life course” that includes repeated deep phenotypic data and biosample collection, with whole genome sequencing on a subset of the cohort [16]. Participants comprise a cohort of offspring born to pregnant women recruited between April 1991 and December 1992 in Bristol, UK, their parents, grandparents, siblings and most recently their own offspring [16]. The birth cohort (now young adults) are also referred to–and frequently refer to themselves–as Children of the 90s (Co90s). Alongside their families, participants have been followed through a series of ongoing data collection time points involving questionnaires and clinical assessment. Sub-studies involving smaller samples are ongoing, including RbG studies. In all sub-studies it is ALSPAC administrators who circulate invitations to existing participants on behalf of research teams within and beyond ALSPAC, with invites arriving on Co90s letterhead signed by ALSPAC principal investigators.

Measures are taken to ensure cohort participants are not overburdened with study invitations. To conduct research involving cohort participants, sub-study researchers work with ALSPAC administrators to ensure all recruitment materials meet established standards and protocols. Following the principles and methodologies of Responsible Research and Innovation [17,18], ALSPAC participants are engaged in the approval processes for all research conducted within ALSPAC and take part in governance decisions. Each study application is reviewed by the Original Cohort Advisory Panel (OCAP), a committee of ALSPAC participants who provide input and advice on study design, methodology and acceptability based on their expertise as participants. Study applications are then submitted to the ALSPAC Ethics and Law Committee (ALEC) for final approval. ALSPAC participants comprise half the membership of the ALEC, which is currently chaired by a parent participant and deputy chaired by one of the Original Cohort. It was previously chaired by one of the authors (MJM).

Genome-wide common single nucleotide polymorphism data are now available for over 18000 cohort participants, including a sub-sample of approximately 1800 participants for whom there is complete genome sequencing [19]. Since 2010, RbG studies of genetic variants associated with topics such as sleep, body mass index (BMI) [20], schizophrenia, smoking behaviour and platelet function [21] have been conducted in ALSPAC. ALSPAC maintains a general principle that biomedical information is not disclosed to cohort participants unless there is clear evidence that the benefits outweigh the risks and that three conditions are met: (1) data provide clear, unequivocal evidence of an existing or future health problem; (2) said health problem is amenable to treatment of proven benefit; and (3) the participant has indicated in advance a wish to be informed if such a problem is identified [22]. The method and nature of RbG studies is fully described in ALSPAC recruitment documents but specific genetic variation(s) are not communicated to potential participants.

With no available empirical evidence of the views of the wider ALSPAC participant population, we undertook a qualitative study of participant perspectives on the recruitment of cohort participants into RbG research and the possible receipt of individual genetic information. ALSPAC participants are well placed for RbG studies because they have already been recruited, contact is straightforward and many have already given consent for genetic data to be used for future research. Our study sought both to contribute empirical consideration of RbG recruitment from participants’ perspectives to the scientific literature generally and improvements in specific local ALSPAC governance of the research it approves and undertakes.

## Methods

### Ethics

Ethical approval for this qualitative study was obtained from the Avon Longitudinal Study of Parents And Children, Law and Ethics Committee, University of Bristol (ALEC) (approval reference no. 13341).

### Recruitment and data collection

A purposive sample (i.e. a non-probability sample of participants based on our research objectives) was generated across three categories: (1) general ALSPAC cohort participants who had never participated in an RbG study; (2) participants who had participated in an ALSPAC RbG study; and (3) individuals who had served on one or more ALSPAC committees at which RbG study applications were discussed (e.g. ALEC, OCAP). In May 2016, study invitations were mailed to 200 ALSPAC participants (S1 Table), of whom 74 returned expression of interest forms. Each respondent was followed up at least twice to arrange an interview date and time; 21 did not reply to follow up or were unable to schedule an interview within the data collection timeframe (June to August 2016). The final response rate (26.5%) is reflective of the often mobile and changeable circumstances of these young adults as they transitioned between education and work. All participants who were able to attend an interview (in person or by phone/Facetime/Skype) within the data collection timeframe were included. Interviews continued beyond the point of saturation(the point at which no new themes were forthcoming) [23] in each of the three sub-groups. Our decision to continue interviewing was based on an ethical commitment to respect participants’ wishes to be included as well as a methodological opportunity to evaluate our assessment of saturation. Once we had concluded that no new themes were emerging, important issues were re-emphasised in the latter interviews and additional negative cases (contrasting examples) were elicited. In total, 53 semi-structured interviews were conducted. In keeping with established ALSPAC practice, participants each received a £20 gift voucher and were reimbursed for travel costs where applicable. An interview topic guide was developed, approved by the ALEC, and revised iteratively throughout the interviews [23] (S2 Table). Feedback was also solicited on a leaflet distributed with the invitations explaining the principles of RbG research [24]. Interviews were conducted by two of the authors (FB (n = 20) and JTM (n = 33)), lasted between 14 and 62 minutes, and were audio-recorded and transcribed verbatim with participant’s consent. Resultant transcripts were checked for accuracy and anonymised.

### Methodology and epistemic framework

This paper provides an example of empirical ethics; that is, it is a consideration of ethics based on primary research rather than argumentation drawn from ethical, legal or other normative principles. Empirical ethics, as any research, may be based on a variety of epistemic or methodological assumptions. We take a moment here to describe those roots so the reader is better placed to judge our interpretations and interpret our findings. The methodology used for this study was constructivist interpretivist: “particular actors, in particular places, at particular times, fashion meaning out of events and phenomena through prolonged, complex processes of social interaction involving history, language and action” and “that to understand this world of meaning one must interpret it” [25]. Following Mol [26,27], we also start from a premise that the individual subject of ethical consideration is multiple rather than singular or plural. The research participant imagined for informed consent is typically an autonomous, rationale, choosing individual who will, with sufficiently accurate and unbiased information, make decisions about their involvement in research based on their preferences and values. This is not so much an untrue description as it is overly simplistic for analytic purchase. A research participant might instead be considered plural, holding different perspectives based on background and experience that in turn shape that person’s values, preferences and decisions. But Mol argues that as persons we inhabit many possible personhoods: we can be both parent and child, teacher and learner, leader and team member. Indeed, we may be all of these persons (or subjects) in different settings. Each different way of being a person (or subject) is shaped by what we expect to do or be and by what is expected of us. We can see these as being different subjects, or, less ontologically confronting, as persons taking on different subject positions inhabited based on circumstance and which are situated and relational; that is, they occur in specific social and geographic settings and in relationships with other persons.

While there is much in common, there is an important distinction between understanding the world as inhabited by plural or by multiple subjects. Seeing the subject as plural understands individuals whose views may differ from one another but for whom there is an internal coherence of perspective. Seeing the subject as multiple understands individuals as each potentially inhabiting many possible subject positions, at different moments and in different settings. For the purposes of analysis here, we consider our research participants as capable of holding more than one subject position and that these are not always apparently or necessarily coherent. This is the epistemic basis for the interpretations we make of the data collected; as such, our approach is a necessarily constructionist rather than realist epistemology.

### Data analysis

Interviews were coded thematically using the constant comparative approach [23] and following the steps provided by Braun and Clarke [28] for organising analysis without imposition of a specific epistemic framework. This approach involves researchers familiarising themselves with the data corpus (e.g. the interview data in this study), generating initial codes and then developing, reviewing and defining themes. Having identified our themes using this approach, we undertook an interpretive analysis of them based on the epistemic framework outlined earlier. The findings presented here are necessarily one of a number of possible interpretations; that is, ours is not a post-positivist analysis that assumes a singular interpretation [29]. Nor is it exhaustive: the wealth of data collected in qualitative research enables multiple questions to be posed [29]. In order to warrant our interpretations, we present verbatim extracts from the data and offer thick descriptions [30] of these interpretations to enable the reader to judge the soundness of our interpretations [31]. We also undertook to enhance the quality and credibility of our analysis following Patton [32,33] by testing rival explanations, searching for negative or disconfirming cases, triangulating through multiple analysts, and challenging the credibility of the researcher(s).

This epistemic framework and deep familiarisation with the interview data (which included listening to the audio recordings throughout the data collection period) allowed the authors (JTM, FB, MJM) to revise the topic guide iteratively and provided the basis for them reaching decisions about saturation. To allow FB to complete the requirements for a Master’s research project, she conducted and coded 20 of the first 21 interviews, identifying and exploring preliminary themes under the direction of MJM and FB’s project supervisor (see Acknowledgments). This work was completed prior to data analysis of all 53 interviews for this paper, which involved JTM, FB and MJM familiarising themselves with the data by repeatedly and independently reading all of the transcripts and listening to the audio-recordings. Then, based on joint discussions among all three, JTM developed a coding frame for analysis and coded the transcripts using NVivo 11 software. MJM undertook additional checks on coding and interpretation. The results were considered in relation to FB’s earlier work and as such offered a means to assess inter-rater reliability. Throughout the main analytic process, disconfirming cases (instances where participant perspectives ran counter to the developing interpretations) were sought to challenge emergent constructs, evaluate alternate explanations and refine resultant interpretations. Lastly, the final text was reviewed against the coded data to ensure accuracy. In keeping with standard qualitative practice, brief excerpts of participants’ comments are provided in support of our analytic findings. All individuals quoted were pseudonymised specifically for this paper, nominated alphabetically throughout and indicative of gender only. To protect participant confidentiality, the qualitative data in this study are accessible through managed access under the ALSPAC Access Policy, Version 8.0, May 2018. These restrictions form part of the ethical agreements made with study participants and approved by the Avon Longitudinal Study of Parents And Children, Law and Ethics Committee, University of Bristol (ALEC) (approval reference no. 13341). Applications for access should be made to the ALSPAC Data Access Committee through its online portal: https://proposals.epi.bristol.ac.uk/. Information about the ALSPAC Access Policy and process is available at (http://www.bristol.ac.uk/alspac/researchers/access/).

## Results

Of the 53 participants interviewed, 29 were female and 51 has been enrolled in ALSPAC continuously since birth. We include no further participant characteristics in order to maintain the privacy of the individuals involved. This paper identifies three key participant perspectives about recruitment, the return of research results generally and the return of results in relation to RbG research in particular.

### Perspectives on participation

Participants all reported very high levels of trust and engagement in both the administration and scientific goals of the ALSPAC project. They frequently expressed satisfaction at having played an important role in the advancement of medical research and discovery of new treatments. When asked how they decided whether to accept new research invitations from ALSPAC, almost all reported their default choice was to participate. Many also indicated they gave only limited consideration to the research topic under consideration or the information provided in recruitment documents.

*Because I’ve been part of Children of the ‘90s all my life*, *I kind of just don’t really think about it*. *I just do it*. *It’s just part of something that I do now*. *I tend to just do whatever I can to help*, *really*, *because it’s such a big study and they’ve found out so much information that I think if I can help then*, *yeah*, *so I just do it*. [Alice]

Few participants could recall having declined a study invitation. Where they could, only two reasons were given: practical barriers (e.g. time restrictions; geographical distance) or personal dislikes (e.g. drawing blood; breast examinations).

Overall, participants expressed limited interest in knowing why they had been invited. While most did not feel it was important to know the reason for their recruitment, a few indicated such information could be interesting, though not knowing would not deter participation.

*I’m not too bothered about why*. *It’s sometimes nice to know just out of interest*, *but it wouldn’t influence whether I said yes or no*. [Belinda]

A few participants identified concerns around participation in research, either ‘front end’ issues (e.g. gauging potential risk; agreeing with a study’s aims) or post-study concerns (e.g. potential return of unwelcome adverse findings). Again, such concerns were almost never expressed as barriers to participation.

*I mean there is always that worry that*, *you know*, *something might be wrong with you and you might never find out even if all the data is collected*. *I guess I would be upset to find out later that I had some kind of terrible disease which I could have known about if I had been told why I was invited to something*. [Cathryn]

Many participants used their established trust and faith in ALSPAC to ‘shorthand’ decision-making about recruitment. Participants also frequently blurred any distinction between ALSPAC data collection events (known as Focus Days) and sub-studies conducted by outside researchers. This finding did not lead us to conclude that participants were unable or unwilling to think critically about recruitment and participation; rather, we found most simply did not perceive a need to do so. The exception were participants who had served on ALSPAC panels and committees. While equally expressive of trust and faith in ALSPAC, these individuals appeared to engage more thoroughly with study invitations, even if their decision to participate was similar to other participants.

### Perspectives on genetics

While our study did not measure participants’ knowledge of genetics, the interview data suggested most had a basic high school level of understanding, with a smaller number either quite knowledgeable or largely uninformed. Participants typically found the level of explanation in an ALSPAC leaflet about RbG research [24] (written at high school level knowledge and included with the study invitations) was accessible and would likely be so for other cohort participants. Where individuals reflected on genetics in their interviews, they typically used terminology expressing curiosity or interest (*I’d just love to know really what’s different about me and how that maybe affects me compared to other people*. [Deborah]) or perceived certainty (*Your DNA is essentially what you are built of*. *There is no escaping your own DNA*. [Edwin]).

Given the iterative nature of the interview process, only the final 14 participants were asked specifically how much they reflected on their genetic make-up in everyday life. Few reported doing so to any extent; personal genetics was expressed more as a “*backburner thing*” [Fred] of limited interest. In itself, this finding was not surprising since the participants’ age and general circumstance (i.e. mid-20s, still establishing careers and personal relationships) would not suggest any pressing need to reflect regularly on genetics. The perspective of one participant–a new parent–did hint, however, that this could change.

*To be honest*, *I never used to really think about [my genetic make-up]*. *However*, *I do think about it more now*. *It’s something that*, *obviously with my little one*, *it’s something that you do think about and you do kind of wonder how it all works*. [Alice]

As regards genetics and RbG research, few participants besides those who had served on ALSPAC panels and committees indicated much knowledge of this recruitment technique. Because over half of our sample involved individuals who had already participated in an ALSPAC RbG study, this finding illustrated the known tendency of research participants to forget the details of research in which they are involved, specifically the information in consent forms and participant leaflets [34,35]. This may also suggest, however, that previous ALSPAC recruitment materials could have been even more clear and concise (even though these had been reviewed by participants in advance).

RbG research as discussed was generally seen in either positive or neutral terms (“*a lot more efficient*” [Fionnuala]; “*just another study*” [Georgina]). Few participants felt their decision to participate would be influenced by knowing they had been invited based on a genetic variation. Those who thought it might be also stated such information could make them *more* likely to enrol. The authors caution against interpreting this finding as evidence of unconditional acceptance of RbG research because some participants also incorporated suggestions of unease or hesitation into their observations of genetics-based research. For example, when asked to reflect on possible disadvantages of RbG research, one participant observed:

*I suppose [RbG studies] might reveal things that people don’t want to know*, *including negative outcomes*. *I suppose people don’t want to know that*. [Joanne]

Another individual who knew she had already participated in an RbG study expressed ongoing concerns:

*I still think about it now because I still think–I guess I would have liked–I’m someone who would have liked to have known which group [I was in]*. *But there’s no way of telling me because then it would affect the study if I knew or it could affect it*. *But I still think*, *“Oh*, *I wonder which group I was part of*?*” because I wasn’t actually told*. [Heather]

While participants largely reported being unconcerned about RbG research, experiences such as Heather’s could increase as RbG recruitment becomes more common. Though not yet evident among our participants at their life stage (excepting the one parent-participant), if cohort participants begin to reflect more critically about on their genetics (e.g. once they have or are planning to have children) this may change. Individuals who once saw little reason to question involvement in RbG studies might develop a stronger interest in doing so.

### Perspectives on the return of research results

There was near universal awareness and acceptance of ALSPAC’s practice to return biomedical information to cohort participants only under the general principles discussed earlier. Participants expressed little expectation they would receive information about themselves.

*I’ve never questioned it [information provision in ALSPAC] because sometimes you’ll come away and you’ll have nothing or they can give you a scan or something and it’s nice to come away with something. But I suppose it’s not about me. I mean, it’s more about the overall picture. So, I never expect to receive anything. It would be really interesting but I understand it’s such a large scale thing that they couldn’t, you know, analyse everyone and give everyone individual feedback, and I understand that, so it’s not a thing [laughs].* [Isabelle]

Such data led us to conclude that ALSPAC’s non-disclosure policy was widely accepted for three reasons: it had been communicated repeatedly and clearly; individuals trusted ALSPAC to act if significant and remediable results were found; and participants viewed their role in ALSPAC primarily as data providers. Regarding communication of research findings more generally, most participants reported being only moderately interested in the outcomes of ALSPAC studies and were happy to receive such information via occasional newsletters. When presented with the possibility of using technology to ‘push’ personalised digests of findings of specific interest (e.g. asthma), few saw much appeal. Similarly, there was little interest in receiving routine individual results (despite many participants recalling fondly having received souvenir copies of scans and such as children).

While our participants did not generally express any strong concerns about the return of research information, the authors did identify two matters of note. First, ALSPAC was very much expected to observe its current policy. Participants articulated this expectation either as an ethical obligation or as a benefit arising from being a cohort member. This was a general expectation and not restricted to RbG studies or genetic information.

*I went to my last Focus group [a routine data collection point, not an RbG study] and then I got a phone call from the [ALSPAC] doctor a week later because my blood results weren’t quite right. And they advised me to go to the doctor’s and have a repeat. And it was all fine in the end, but I think that was really good of them to call me–to let me know–because if I hadn’t have gone to Children of the 90s and hadn’t had that blood test, then it might have been something more serious and then I would have never have known about it.* [Jackie]

The second concern noted among participants about the return of information related specifically to RbG research. During the interviews individuals were presented with a hypothetical scenario in which an RbG study identified a link between a genetic variation and an increased likelihood of developing type 2 diabetes in middle age. Most participants felt ALSPAC had a duty to tell them both the outcome of such a study and whether they personally carried the variation in question (either at the time of recruitment or following the study). The reasons cited turned less on ethical obligation or participatory benefit and more on a belief that health threats in the future might potentially be mitigated through behaviour change.

*To be honest, I don’t know much about … type 2 diabetes, so if it was possible for me to prevent it by controlling my external factors then, yes, they should let me know. I mean, I think either way, yes, they should let me know. But if there was a way to prevent it, then it’s more important for them to let me know so I could do something about it and I’d have more control over it.* [Katy]

Finally, participants were asked to consider the relative cost of returning individual results, especially given an anticipated increase in RbG research. While individuals expected ALSPAC to return adverse findings of clinical significance (such as the hypothetical study on type 2 diabetes), most preferred funds be spent on research if the mechanics of returning results proved too costly.

*…in an ideal world we’d get both, so without having to take any budget or resources from the study or publication of research and be able to bring in counsellors or whatever, and be able to relay that information back on to me, that would be the ideal world. But if it’s not the case and it’s not feasible–if it’s not financially viable–then I wouldn’t have any gripes, I suppose. It wouldn’t stop me from wanting to do the study.* [Liam]

This finding suggested that participants’ perspectives on the return of results could become more complex and dynamic as the number of RbG studies increases. Such a change might challenge current ALSPAC policy on returning results if participants situate the health consequences of such findings far enough into the future that individuals feel they can effect change and prevent negative outcomes. In such a situation, cohort members may no longer be willing to prioritise research over returning results.

## Discussion

Our study demonstrated that participants’ perspectives on RbG research could only be fully understood within the context of their long term and ongoing experience within ALSPAC. Capturing perspectives on RbG research required acknowledging the expertise participants had acquired as data providers over 20+ years and the impact their relationship with ALSPAC has had on engagement with the research. Like Michie’s [8] Cystic Fibrosis patient participants, ALSPAC participants demonstrated societal solidarity [13], seeing themselves as contributing to a greater good. They also understood this contribution to be reciprocal: participants appreciated learning something about themselves and expected ASLPAC to act in their interests as the need arose. Our results underscore the importance of learning from the experience of study participants when addressing emergent governance challenges posed by new methodologies. Although participants in our study seemingly expressed limited interest in knowing the reasons for their recruitment or the findings of research, their attitudes spoke to both the ongoing advantages and limitations of their close working relationship with ALSPAC. Indeed, the strength of trust in ALSPAC’s ‘duty of care’ role provides an important understanding, namely that any such perception or expectation arguably confers on the study a higher responsibility towards its participants than in a study where engagement is less strong. Despite an explicit commitment by ALSPAC not to over-burden participants, such factors could leave participants potentially vulnerable to over-recruitment or foster a default willingness to consent. Moreover, in early 2018 ALSPAC introduced a two-step approach to address some of ethical challenges when recruiting for RbG studies. This change was developed by working with participants and members of the ALEC and was undertaken cognisant of this study’s findings. The new approach recognises that there can be sensitivity through invitation alone to an RbG study and that ALSPAC would not knowingly disclose information about individual carriage of a genetic variation during the recruitment process. All participants are being contacted to communicate the particulars of RbG research and to explain how genetic risk works. This is being accomplished using the leaflet included in our study [24]. Participants are then being asked whether they are in principle open to receiving invitations for this type of research; those who are not can then opt out.

While the ethical issues facing RbG research are closer to those of population-based genetic research than family studies [36], such an approach is inextricably linked to the issue of disclosing individual results [4]. There is undoubtedly a distinction between genetic data of clinical utility and data commonly used in epidemiological research, but differences in the implications of such categories for informed consent and ethical study conduct are often blurred [37]. Where a variant is associated with a serious or stigmatising condition, a decision is needed about whether it is in the best interests of potential participants to conceal that condition. This decision cannot be made without the input of participants themselves, either through their routine involvement in study governance or through empirical studies such as this one. But such input must not be deemed sufficient for all time: participants’ perspectives and needs are dynamic and can evolve. In a project with strong participant engagement, participants must be engaged fully in the determination of what counts as open communication and the value of individual genotype disclosure.

We have attempted in this paper to contextualise the central tension between avoiding the possibility of participant harm through revealing unwanted or misunderstood information, and avoiding deception when explaining recruitment into RbG studies [15]. To date only a limited number of studies have attempted to assess the effects of incorporating genetic information in intervention designs [38,39]. While some studies demonstrated the strong social and cultural determinants of those influences in the face of information about genetic susceptibility [40], evidence on the impact of using genetic data within recall studies is lacking. Given the qualitative nature of this study, the analytic conclusions drawn are situationally generalisable to other longitudinal birth cohort studies like ALSPAC. Beyond this, our findings are theoretically generalizable [41,42]; parallels may be drawn more widely in relation to ethics practices and RbG research, at least until further empirical work has been reported. For example, population-level biobanks such as UK Biobank and the All of Us study face ethical conundrums similar to those examined in this paper. Although population-based biobanks may not be centred around a common experience in ways which have been shown to produce feelings of solidarity in disease-based biobanks [8] other aspects of solidarity and co-production may be present; participants in all longitudinal research pass through shared life milestones and developmental stages. Our findings suggest that whether recruited at birth or in adulthood, some individuals may come to identify strongly with their study and see themselves as part of the research team, particularly as participant engagement initiatives become more commonplace. We argue that there are already common expectations for truthfulness and the option to be told (or not) of critically important information like incidental findings. What remains to be determined is whether there exists a sliding scale of expectation by study type about what is communicated upfront or fed back post-study.

Seven recommendations emerged from a consensus workshop about recruitment to studies based on genotype [15] offering a good starting point for other studies. All were followed by ALSPAC: (1) participants be made aware of potential for re-contact; (2) participants have a choice about whether to be re-contacted; (3) re-contact be made by a known person or entity; (4) recruitment be based on the biobank’s own processes; (5) thresholds for the return of incidental findings be considered differently to the return of genetic information during recruitment; (6) genetic research information offered in the context of RbG recruitment should not leave participants uninformed about the study’s purpose; and (7) approaches to RbG recruitment be determined in consultation with ethics committees [15]. Our study highlights how such recommendations are best seen as reflective of one point in an evolving, iterative relationship between a longitudinal study and its participants, one impacted regularly by shifting social norms and technological advances. Such a perspective is particularly true of the sixth recommendation, which must be tempered by the nature of the condition associated with a variant (e.g. treatability, stigmatisation), and the seventh recommendation, which is better expanded to include participants as well as ethics committees.

Furthermore, studies like ALSPAC that have a strong engagement ethic must continue to engage not only researchers and ethics committees but also their participants, who hold a separate and unique form of expertise. ALSPAC can draw not only from its ethics committee but from its participant panels and qualitative studies such as this one. Between them, innovative approaches can be developed to provide information to participants in formats offering multiple levels of explanation; advance creative mechanisms for communicating information on increasingly complex topics; and offer opportunities for participants themselves to explore and critique the ethical implications of new study designs. Studies such as ALSPAC must be mindful that in the context of a strong and established study-participant relationship, the inclination of participants can be to decide first and ask questions later when considering study invitations. Attention to such understandings will underpin governance practices and help ensure they remain fit for purpose. Governance practices which centrally include participants in decision making processes are, arguably, best suited to developing appropriate, trustworthy and credible safeguards and oversight of cohort and biobank research.

*Limitations of the study*. While our findings may be suggestive of perspectives found among other longitudinal studies, they are not likely transferable directly to other types of biobanks with one-off collections (e.g. disease or tissue-based biobanks). Our sample was self-selected and as such we may have heard disproportionately from individuals interested in the topic and/or open to discussing a potentially sensitive subject matter. Nonetheless, this empirical study demonstrates that doing so is necessary to understand study participants’ perspectives in terms of their wider experience of involvement in a biobank.

## Supporting information

S1 Table Participant recruitment. (DOCX)

S2 Table Interview topic guide. (DOCX)
