# Supplementary material for: The ethics conundrum in Recall by Genotype (RbG) research: Perspectives from birth cohort participants
Source: PLoS One. 2018 Aug 16;13(8):e0202502. doi: 10.1371/journal.pone.0202502 (PMC6095592; doi:10.1371/journal.pone.0202502)
Supplement: S1 Table — (DOCX) [file pone.0202502.s001.docx]

**The ethics conundrum in RbG research**

Minion, Butcher, Timpson, Murtagh

**Table 1: Participant Recruitment**

|  | Total | General | RbG | Advisory | Male | Female |
| --- | --- | --- | --- | --- | --- | --- |
| Invited | 200  (100%) | 100  (50.0%) | 80  (40.0%) | 20  (10.0%) | 83  (41.5%) | 117  (58.5%) |
| Interviewed | 53  (25.5%) | 19  (35.9%) | 30  (56.6%) | 4  (7.6%) | 24  (45.3%) | 29  (54.7%) |

General = active members of the general Co90s cohort who had not participated in a RbG study

RbG = active cohort members who had participated in a Co90s RbG study regardless of whether they understood or recalled the study as having recruited by genotype

Advisory = cohort members involved in ALSPAC panels and committees
