# Supplementary material for: The ethics conundrum in Recall by Genotype (RbG) research: Perspectives from birth cohort participants
Source: PLoS One. 2018 Aug 16;13(8):e0202502. doi: 10.1371/journal.pone.0202502 (PMC6095592; doi:10.1371/journal.pone.0202502)
Supplement: S2 Table — (DOCX) [file pone.0202502.s002.docx]

**Table S2: Interview topic guide**

| Participating in ALSPAC research | - How do you decide whether to participate when invited to a new study? Have you ever turned down participating in a study? (ask for details) - How important is it for you to know why you were invited? Can you think of examples? Can you think of a situation where your decision might be different if you knew why you were invited? - Have you heard about RBG studies before? Do you have any particular views on recruiting participants based on genetic variation? - Did you get a chance to look over the RBG leaflet? If so, how well did it improve your understanding of RBG research? How well do you think other Co90s participants would understand it? How could the leaflet be improved? |
| --- | --- |
| Return of results | - How important is it for you to learn the general findings of a study? How would you prefer to receive such information? Have you ever actively looked for published papers or media reports? - If there was something you were really interested in (e.g. research on asthma), would you like to have such findings fed back? - How important would it be for you to be provided with study results specifically about you? |
| Recall by genotype research | - How much do you think about your genetic makeup and its impact on your life? - If you were invited to be part of a RBG study, would you feel any different about being in it? What if you were told you were being invited because you carried a particular genetic variation? - Let’s say you take part in an RBG study that discovers a particular genetic variation leads to a significantly increased likelihood of developing Type 2 diabetes in middle age. Do you think you should be told if you have the variation? How do you think you would feel if you were told? - Are there some research findings you would expect to be told about but not others? Do you think you would feel any different about receiving general findings from a RBG? - If Co90s were to begin feeding back more individual results, doing so would likely require additional clinical, counselling and other staff resources. This could mean funds would need to be diverted away from other research. How might this impact your views about feeding back findings? |
| General questions | - What has it been like being part of the Children of the 90s? Do you see yourself taking part for another 25 years? - How much do you think participants should be involved in helping Co90s make decisions about how the main study is run, what sub-studies are approved and where Co90s is headed in the future? Is there anything about which participants should have a say (e.g. commercialisation)? - If Co90s suddenly came into a large pool of money to do new research, what suggestions might you have for them on what to investigate? |
| Conclusion | - Do you have anything else you’d like to include in the interview before we finish? - Is there anything you think we should have asked but didn’t? |

Qualitative research is by nature an iterative process. Topic guides develop and are revised throughout the data collection process as questions are reviewed and evaluated; questions within the topic guide are tailored to the interests and experiences of the participant.
